# A New Approach to Guided Wave Ray Tomography for Temperature-Robust Damage Detection Using Piezoelectric Sensors

**DOI:** 10.3390/s18103518

**Published:** 2018-10-18

**Authors:** Dan Li, Ming Shi, Feng Xu, Chengcheng Liu, Jianqiu Zhang, Dean Ta

**Affiliations:** 1 Research Center of Smart Networks and Systems, The Department of Electronic Engineering, Fudan University, Shanghai 200433, China; lidan@fudan.edu.cn (D.L.); mingshi13@fudan.edu.cn (M.S.); xf@fudan.edu.cn (F.X.); tda@fudan.edu.cn (D.T.); 2 Institute of Acoustics, School of Physics Science and Engineering, Tongji University, Shanghai 200092, China; chengchengliu@tongji.edu.cn

**Keywords:** guided wave, ray tomography, TOF, temperature compensation, structural health monitoring, piezoelectric sensors

## Abstract

In this paper, a new approach to guided wave ray tomography for temperature-robust damage detection with time-of-flight (TOF) temperature compensation is developed. Based on the linear relationship between the TOF of a guided wave and temperature, analyses show that the TOF of the baseline signal can be compensated by the temperature measurement of the inspected materials without estimating the temperature compensation parameters. The inversion is based on the optimization of the TOF misfit function between the inspected and compensated baseline TOFs of the guided waves, and is applied by the elastic net penalty approach to perform thickness change mapping in a structural health monitoring (SHM) application. Experiments that are conducted in isotropic plates by piezoelectric sensors demonstrate the effectiveness of the proposed method. According to the results, our approach not only eliminates the artefacts that are caused by a temperature variation from 25 °C to 70 °C but also provides more accurate and clearer imaging of damage than conventional ray tomography methods.

## 1. Introduction

Damage of industrial infrastructure, aircraft, bridges, pipes, and rails is a significant problem that involves various safety issues [1]. Ultrasonic guided waves, which are used as a reliable tool in structural health monitoring (SHM) for detecting and monitoring damage, have received considerable attention because these waves can travel over long distances with less signal loss and thus can cover large monitoring areas [2,3,4,5]. In an SHM system that uses guided waves for damage detection, ultrasonic transducers can be permanently attached to plate-like or pipe-like structures. Each transducer, in turn, generates guided waves, which propagate through the structure and interact with damage, and the remaining transducers record the receiving wave motion. These recorded signals can be processed to generate an image with the locations of damage via many algorithms such as travel-time tomography [6,7,8,9], diffraction tomography [10,11], the hybrid algorithm for robust breast ultrasound tomography (HARBUT) algorithms [12,13] and the full waveform inversion (FWI) method [14,15,16,17,18]. Damage in inspected materials can cause changes in the guided wave waveforms. However, temperature variations of materials can also greatly affect the amplitudes and phases of guided waves, especially those of baseline subtraction signals [19,20]. In other words, changes in temperature can result in changes in the guided wave waveforms. The tomography algorithms that are reported in [6,7,8,9,10,11,12,13,14,15,16,17], which image damage mainly based on the differences between the inspected and baseline signals, cannot distinguish whether the changes in the waveforms are caused by temperature variations or damage in the detected material.

To mitigate the effect of temperature on guided waves, a considerable amount of research has been undertaken on temperature compensation of guided waves in the last decades. The baseline signal stretch (BSS) method [21,22], optimal baseline selection (OBS) method [23,24], and hybrid algorithms that combine the BSS and OBS methods are the most commonly used approaches [25,26,27]. The BSS method compensates for the effect of temperature by modifying the waveform of the baseline signal to make it match the inspected signal in terms of the mean square deviation. However, it is only suitable for a temperature variation range of a few degrees [20,21]. To accommodate a larger temperature variation range, the OBS method uses criteria that are similar to those that are used in the BBS procedure to select a best-matched waveform from the baseline signal database, which contains signals that were measured at various temperatures. This method requires many baseline signals that have sufficiently low post-subtraction noise levels for various temperatures. In various situations, it may be impossible to acquire a baseline signal database with the required temperature resolution. The hybrid algorithms, which combine BBS and OBS, can achieve temperature compensation with a reduced number of baseline signals. This method selects a best-matched waveform from a baseline signal database that has low temperature resolution. The best-matched waveform is then used to compensate the inspected signal via the BBS method. In addition to these algorithms, other temperature compensation methods have been proposed in the literature, such as combinations of the OBS method and the adaptive filter algorithm [28] and of the Hilbert transform and the orthogonal matching pursuit algorithm [20]. As discussed above, most temperature compensation methods model the differences between the baseline and inspected signal waveforms that are caused by the temperature variations as an amplitude scale factor and a phase shift. Once the amplitude scale factor and the phase shift have been estimated, the guided wave waveform can be obtained via temperature compensation. These methods are limited by the estimation accuracies of the amplitude scale factor and the phase shift, which depend on the length of the time window that is used in the temperature compensation parameter estimation. In addition to altering the waveforms of guided waves, temperature can affect the piezoelectric transducer [29]. However, the effect of temperature on transducer performance is significantly less than the effect of temperature on guided wave propagation [30].

In this paper, we propose a new approach to guided wave ray tomography for temperature-robust damage detection. Using the linear relationship between the TOFs of guided waves and the temperature variations, it is shown that the TOFs of the baseline signal can be compensated by the temperature measurement of the inspected material without estimating the temperature compensation parameters. After that, an optimization problem that is based on the difference between the inspected and compensated baseline TOFs of the guided waves is formulated via an elastic net penalty approach, and is resolved to image the damage in an SHM application. To evaluate the effectiveness of our approach, experiments were conducted in isotropic plates using piezoelectric sensors. The experimental results demonstrate that our approach can eliminate the artefacts that are caused by a temperature variation from 25 °C to 70 °C. They also show that more accurate and clearer imaging of damage than that from conventional ray tomography methods can be achieved.

The remainder of this paper is organized as follows: in Section 2, a temperature compensation method that is based on the TOF of guided waves is presented, and a TOF difference model for damage monitoring based on the differences between the inspected and baseline TOFs of the guided waves is proposed. Using our model, an elastic net penalty approach is used to perform damage imaging via ray tomography. In Section 3, the experimental setup for monitoring the health of an aluminum plate is introduced. The experimental results, which are used to demonstrate the correctness of the proposed method, are reported in Section 4 and discussed in Section 5. Finally, conclusions are presented in Section 6.

## 2. Method

### 2.1. Temperature Compensation Method for TOF

Guided waves refer to waves of strain and stress that propagate along a plate or a pipe with the traction-free boundary condition. Classified according to the displacement distribution in the propagation direction, there are two categories of guided waves: symmetrical and anti-symmetrical modes [31,32]. The A0 guided wave mode is a fundamental anti-symmetric mode of guided waves. Prior to temperature compensation, the TOFs of guided waves are assumed to have been estimated based on the sampled data. The envelope of the A0 guided wave mode is extracted using the Hilbert transform of the received signal. Then, the TOFs of guided waves are determined based on the maxima of the envelope.

It has been demonstrated in [28] that the TOF of a guided wave is linearly related to the temperature, which means that the TOF difference of two guided wave signals that are obtained at different temperatures is proportional to the temperature difference. As a result, one can express it as:

(1) $$\frac{t_{2}-t_{0}}{t_{1}-t_{0}}=\frac{Tem_{2}-Tem_{0}}{Tem_{1}-Tem_{0}}$$

where *Tem*_0_, *Tem*_1_ and *Tem*_2_ denote three temperature measurements of the inspected materials and *t*_0_, *t*_1_ and *t*_2_ represent the TOFs of the guided waves at temperatures *Tem*_0_, *Tem*_1_ and *Tem*_2_, respectively. The TOF shift of guided waves that is caused by temperature can be compensated as follows: According to Equation (1), the TOFs of guided waves at two temperatures and the corresponding temperatures should be measured prior to temperature compensation. Assume that the baseline signal is obtained in an undamaged inspected material at temperature *Tem_b_* and the TOF that is extracted from the baseline signal is denoted as *t_b_.* The reference signal is obtained in the same undamaged inspected material at temperature *Tem_r_* and the TOF that is extracted from the reference signal is denoted as *t_r_*. When the temperature varies, the baseline TOF is also changed. The baseline signal’s TOF, namely, *t_b_*, is compensated according to the temperature measurement *Tem_c_* of an inspected material based on Equation (1); this can be expressed as:

(2) $$t_{bc}=\frac{Tem_{c}-Tem_{b}}{Tem_{r}-Tem_{b}}\left(t_{r}-t_{b}\right)+t_{b}$$

where *t_bc_* is the baseline TOF at temperature *Tem_c_*. If there is no defect in the sensing path, *t_bc_* will be equal to *t_c_*, which represents the TOF that was extracted from the inspected signal that was obtained at temperature *Tem_c_*. In contrast, when the inspected signal has passed through a defect, *t_bc_* is not equal to *t_c_* and the TOF difference between *t_c_* and *t_bc_* is not 0. Therefore, when the inspection of plate-like material damage begins, the inspection temperature *Tem_c_* is measured first. The baseline TOF at temperature *Tem_b_* is compensated by the inspection temperature *Tem_c_* based on Equation (2). After that, the TOF difference between the compensated baseline TOF and inspected TOF is used for damage imaging. In this way, the effect of temperature on damage imaging can be eliminated and Equation (2) is viewed as a temperature compensation method for TOF.

### 2.2. Temperature Compensation Model for Ray Tomography

Based on the proposed temperature compensation method for TOF, a temperature compensation model for ray tomography is developed. Assume that there are two transducer arrays with *M*-element transmitters and receivers that are mounted on a detected plate-like structure. The transducers in the transmitter array are individually used to transmit the guided waves, while all the receivers are exploited to receive the excited guided waves. The TOFs that are extracted from all the recorded data are used to perform thickness mapping.

The monitored area is divided into *N* × *N* grids (*M* ≤ *N*) and each propagation path between the transmitter and receiver is divided into small pieces by these grids. According to ray theory, the TOFs of guided waves in the *M* × *M* propagation paths can be expressed as the following linear Equation [9]:

(3) T=LS

where $T={\left(t_{i}\right)}_{M^{2}\times 1}$ is the column vector of the TOF measurements that are obtained from the received guided wave signals, $S={\left(s_{j}\right)}_{N^{2}\times 1}$ is the column vector of slowness, and $L={\left(l_{ij}\right)}_{M^{2}\times N^{2}}$ is the length matrix of the propagation paths, which are divided into pieces by the grids. Equation (3) is a conventional linear model for ray tomography. By solving Equation (3), one can reconstruct a velocity map of a guided wave and convert it to a thickness map. However, temperature variation is not considered in this model.

Suppose that there is no damage in the monitored plate-like material before its structure is investigated. The column vectors of TOF measurements that are obtained from the received guided wave signals are denoted as the baseline TOF measurements $T_{b}$ at temperature *Tem_b_* and the reference TOF measurements $T_{r}$ at temperature *Tem_r_*. Once $T_{b}$ and $T_{r}$ have been obtained, the system can begin its monitoring work. By inserting $T_{b}$ into Equation (3), one obtains:

(4) $$T_{b}=LS_{b}$$

where $S_{b}$ is the column vector of slowness at temperature *Tem_b_*. Then, the inspection temperature *Tem_c_* is measured, and based on Equation (2), one obtains:

(5) $$T_{bc}=LS_{bc}$$

where $T_{bc}$ is the column vector of the baseline TOF measurements at temperature *Tem_c_* and $S_{bc}$ is the column vector of the baseline slowness at temperature *Tem_c_*. Meanwhile, one may continuously or intermittently obtain the inspected TOF measurements $T_{c}$ that are described by Equation (3). Subtracting Equation (5) from Equation (3), one obtains:

(6) $$T_{c}-T_{bc}=L\left(S_{c}-S_{bc}\right)$$

Let $\Delta T=T_{c}-T_{bc}$ and $\Delta S=S_{c}-S_{bc}$ denote the TOF and slowness differences, respectively. One can rewrite Equation (6) as:

(7) ΔT=LΔS

Compared to Equation (3), Equation (7) contains a temperature parameter, which can be used to perform temperature compensation. Hence, Equation (7) is viewed as a temperature compensation model for ray tomography.

**Figure 1:**
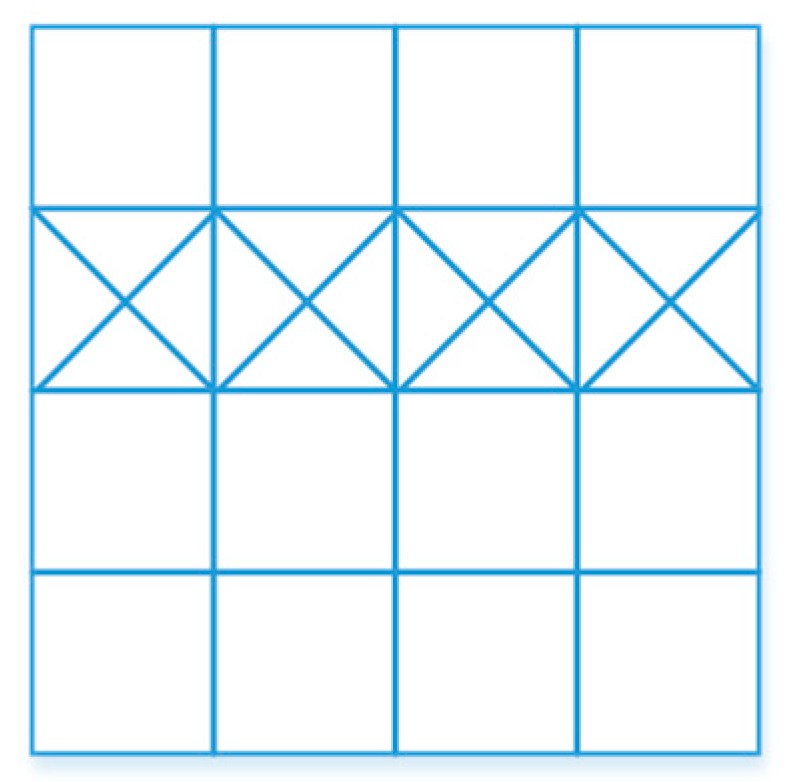
Example of the monitored area, which is divided into 4 × 4 grids.

We assume that the plate damage gradually grows from a small partial thickness. Once a small partially monitored plate has been damaged, the slowness difference of the small partial grids changes. If *K* grids have been corroded, only the values of *K* elements in ΔS are non-zero. Since *K* is much smaller than *N*^2^, ΔS is sparse. In addition, the small partial grids with changed slowness difference are concentrated in the corroded area; hence, the non-zero elements in ΔS will appear continuously. Figure 1 presents such an example, where the monitored area is divided into 4 × 4 grids and four grids have been corroded. The solution of our sparse model from Equation (7) for this example is:

(8) $$\Delta S=\left(\underset{4}{\underset{︸}{0\;0\;0\;0}}\underset{4}{\underset{︸}{\Delta s_{1}\Delta s_{2}\Delta s_{3}\Delta s_{4}}}\underset{4}{\underset{︸}{0\;0\;0\;0}}\underset{4}{\underset{︸}{0\;0\;0\;0}}\right)$$

Four non-zero elements in ΔS appear continuously. If the monitored area is divided into 8 × 8 grids, sixteen non-zero elements in ΔS appear continuously. The grouping effect strengthens as the number of grids increases. Therefore, the solution of Equation (7) has a grouping effect except for the sparsity.

### 2.3. Elastic Net Penalty Approach

Since Equation (7) is also a linear equation, the simultaneous iterative reconstruction technique (SIRT) method [9], which is widely used in ray tomography, can be used to perform damage imaging. However, the solution that is obtained via SIRT is not sparse. Regularization methods can also be used to solve Equation (7) [33]. The solution that is obtained by the regularization methods is sparse; however, the grouping effect of the solutions, which is illustrated in Figure 1, is not taken into consideration.

In [34], a new regularization and variable selection method, namely, the elastic net penalty technique, is proposed. It often outperforms the abovementioned regularization methods, while enjoying similar sparsity of the representation. In addition, it is demonstrated in [34] that the solutions that are obtained via the elastic net penalty technique can cope with the grouping effect. For a nonnegative parameter *α* and a parameter *β* that is strictly between 0 and 1, the elastic net penalty technique solves Equation (7) as follows:

(9) $$min{\left\|\Delta T-L\Delta S\right\|}_{2}^{2}+\alpha \left(\frac{1-\beta }{2}{\left\|\Delta S\right\|}_{2}+\beta {\left\|\Delta S\right\|}_{1}\right)$$

Function $\frac{1-\beta }{2}{\left\|\Delta S\right\|}_{2}+\beta {\left\|\Delta S\right\|}_{1}$ is called the elastic net penalty. The elastic net penalty is the same as the Lasso penalty when *β* = 1. As *β* shrinks towards 0, the elastic net penalty approaches ridge regression. Therefore, the elastic net penalty is viewed as a convex combination of the Lasso and ridge penalties. For β∈(0,1], it is strictly convex [34,35]. Since Equation (9) is a convex problem, if *α* and *β* are specified, the solutions of Equation (9) can be obtained using the CVX toolbox [36,37].

Parameters *α* and *β* are the two key factors in our method. The imaging quality is strongly related to them, especially regarding artefacts. Since *α* is a regularization parameter, the number of non-zero elements in ΔS decreases when *α* increases. In our applications, *α* and *β* are determined in turn. If *β* = 1, *α* ranges between 0.0001 and 0.01. Meanwhile, the optimal value of *α* can be calculated from the L-curve via Equation (9) in [38]. In our experiments, the optimal value of *α* that is calculated via this method is 0.001. Once *α* has been determined as discussed above, *β* can be calculated from the L-curve method when the function $\frac{1-\beta }{2}{\left\|\Delta S\right\|}_{2}+\beta {\left\|\Delta S\right\|}_{1}$ is viewed as a whole. Via this approach, the calculated value of *β* is 0.3.

## 3. Experimental Setup

**Figure 2:**
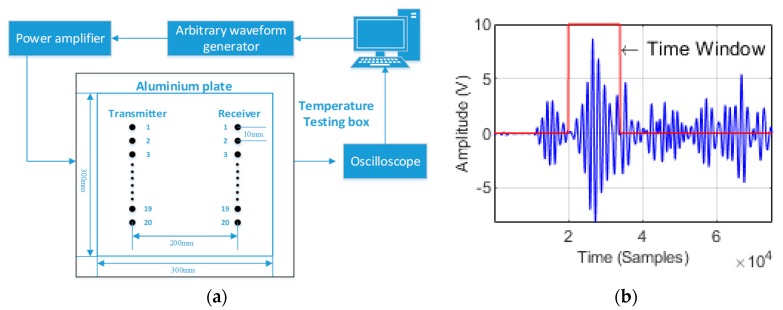

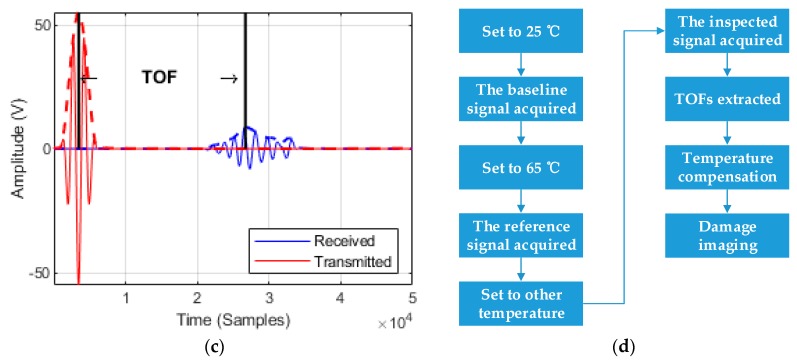
(**a**) SHM system diagram. (**b**) A typical time trace from the experiment within the chosen time window. (**c**) An example of TOF extraction from the transmitter and the receiver. (**d**) A flowchart of the experiment.

The experimental setup is shown in Figure 2a. Experimental measurements are performed on a 300 × 300 × 1.5 m^3^ aluminum plate. Piezoelectric transducers (PZTs) are employed as the ultrasound transducers in our experimental system, where the diameter of each PZT is 5 mm. The central frequency of the transducer is 250 kHz. The transmitter and receiver arrays each have 20 transducers. The transducer spacing is 10 mm for both the transmitter and receiver arrays. The distance between the two arrays is 200 mm. The aluminum plate with transducers is placed into a temperature testing box (custom made by Shan zhi Technologies, Wuhan, China), and its temperature control accuracy is 0.1 °C. The largest aluminum plate that is placed in the temperature testing box is 30 × 30 mm^2^.

During the system operation, the transducers in the transmitter array are used one by one to transmit a 3.5-cycle Hanning-windowed sinusoidal signal, while the receiver transducers are exploited to receive the excited guided waves. For example, when transmitter transducer 1 in Figure 2a is used at a specified time to excite the guided waves, the signal that is received by receiver transducer 1 is recorded by the oscilloscope. At another time, transmitter transducer 1 excites the guided waves again, and the received signal of receiver transducer 2 is sampled by the oscilloscope. This process is repeated until each receiver transducer has operated at least once. To ensure that each receiver transducer measures at the same time, a synchronous signal is outputted each time transmitter transducer 1 excites the guided waves. The oscilloscope sampling of the received signal of each receiver transducer is triggered by the synchronous signal. Then, the remaining transmitter transducers are used one by one to excite the guided waves; the above process is carried out repeatedly. The excitation signal is generated with an initial amplitude of 5 V using an arbitrary waveform generator (33220 A, which was manufactured by Agilent Technologies, Santa Clara, CA, USA) and subsequently amplified to 50 V using a power amplifier (AG1021, which was manufactured by T&C Power Conversion, Inc., Rochester, NY, USA). The received signal is sampled by an oscilloscope (54622A, which was manufactured by Agilent Technologies). The sampling rate of the acquisitions is 250 MS/s. A computer is used to read the received data from the oscilloscope. Figure 2b shows a typical original received signal, where both the transmitter and receiver transducers are the 10th ones. The received signal contains A0 and S0 guided wave modes. The amplitude of the A0 guided wave mode is greater than that of the S0 mode. To ensure imaging quality, a time gating function similar to that in [6] is applied to remove unwanted components, and the A0 guided wave mode is reserved for extracting the TOF. The envelopes of the excited signal and the A0 guided wave mode are extracted using the Hilbert transform. Then, the TOFs of the guided waves are determined based on the maxima of the envelope, as shown in Figure 2c. Next, the TOFs of the guided waves are employed for the inversion.

The flow of the experiment, which is illustrated in Figure 2d, is as follows: The defect-free aluminum plate is placed into the temperature testing box. The temperature testing box is set to 25 °C. The received signals in the defect-free aluminum plate are recorded, and their TOFs are extracted as the baseline TOFs. The temperature testing box is set to 65 °C. The received signals in the defect-free aluminum plate are recorded, and their TOFs are extracted as the reference TOFs. Then, defects are introduced into the aluminum plate, and the plate is placed into the temperature testing box. The temperature testing box is set to a temperature that differs from the baseline and reference temperatures. The received signals in the defective aluminum plate are recorded, and their TOFs are extracted as the inspected TOFs. Our temperature compensation model is used to perform temperature compensation. After that, the defects are imaged via Equation (9).

In our experiment, the wavelength of the A0 guided wave mode is approximately 7 mm. The size of the grid is selected as half of the wavelength. The monitored area is 200 × 200 mm^2^. Therefore, the monitored area is divided into 60 × 60 grids.

## 4. Experimental Results

### 4.1. TOF Versus Temperature Variation Results

**Figure 3:**
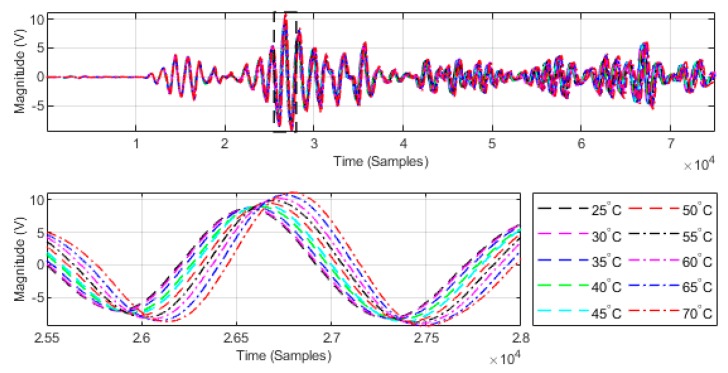
Received signals of the 10th transmitter and receiver transducers at various temperatures in the range from 25 °C to 70 °C.

To verify the relationship between the TOF and temperature, a defect-free aluminum plate is placed into the temperature testing box, where the temperature is gradually increased from 25 °C to 70 °C at a heating rate of 1 °C/min and for every 5 °C step, the temperature is held constant for 40 min. Then, the transmitter transducers are excited one by one with a 3.5-cycle Hanning-windowed sinusoidal signal with a central frequency of 250 kHz, and the receiver transducers record all the received signals using the oscilloscope. Take the 10th transmitter and receiver transducers as an example. The received signals at various temperatures in the range of 25 °C to 70 °C are illustrated in Figure 3.

**Figure 4:**
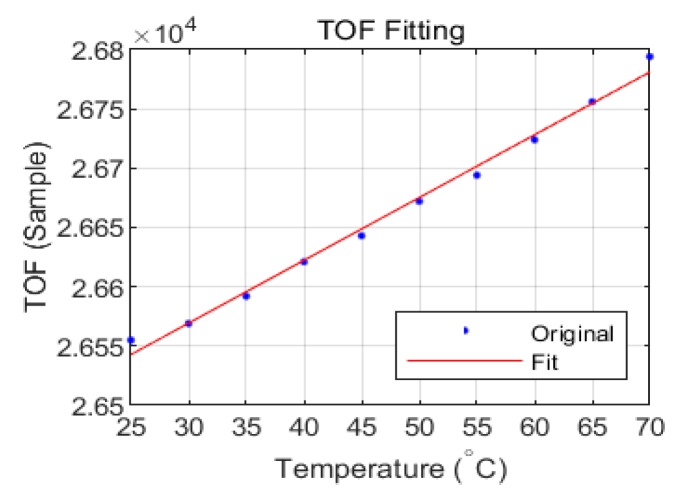
Fit of TOF versus temperature from 25 °C to 70 °C.

The TOFs of the guided waves are influenced by the temperature changes. The TOF increases when the temperature is increased. The least-squares method was employed to fit the TOF as a function of temperature; the result is shown in Figure 4. The linearity of the TOF as a function of temperature with 95% confidence bounds is 0.9923. The result demonstrates that TOF has a strong linear relationship with temperature.

### 4.2. Single Regular Defect

**Figure 5:**
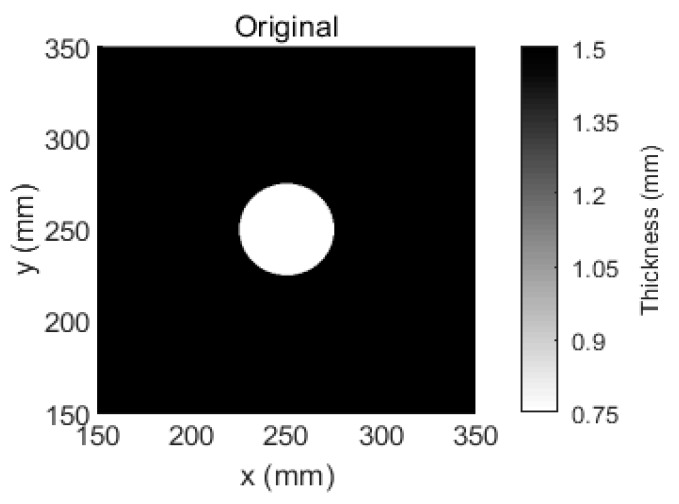
Aluminum plate with a thickness of 1.5 mm and a 50-mm-diameter circular flat-bottomed hole. The thickness loss is 50% within the flaw.

To verify the correctness of the proposed method, an experiment with a single regular defect was conducted. The TOFs that were extracted from the received signals in the defect-free aluminum plate at a temperature of 25 °C were selected as the baseline TOFs and the selected reference temperature was 65 °C. A 50-mm-diameter circular flat-bottomed hole in the center of the same aluminum plate was made such that its thickness loss was 50%, as illustrated in Figure 5. 

**Figure 6:**
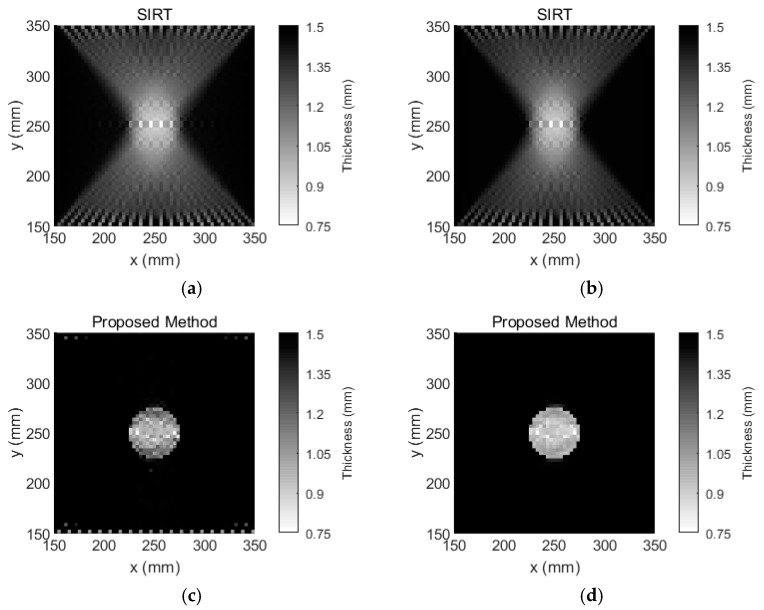
Tomographic reconstruction images for a 1.5-mm-thick aluminum plate with a 50-mm-diameter circular flat-bottomed hole. The thickness loss is 50% within the flaw. (**a**) The tomographic reconstruction image that is obtained via SIRT without temperature compensation, (**b**) the tomographic reconstruction image that is obtained via SIRT with temperature compensation, (**c**) the tomographic reconstruction image that is obtained via our method without temperature compensation, and (**d**) the tomographic reconstruction image that is obtained via our method with temperature compensation.

The aluminum plate was placed in the temperature testing box, and the temperature was set to 60 °C. This temperature is viewed as the inspection temperature. The tomographic images that were reconstructed using SIRT and our Equation (9) are shown in Figure 6. According to Figure 6a,b the spatial resolution of the tomographic reconstruction via SIRT is relatively low. In contrast, according to Figure 6c, the image of the tomographic reconstruction via our Equation (9) is relatively clear, even though temperature compensation was not performed. After temperature compensation, the visible artefacts that were caused by the temperature variations in the image are not observed, as shown in Figure 6d.

### 4.3. Two Regular Defects

**Figure 7:**
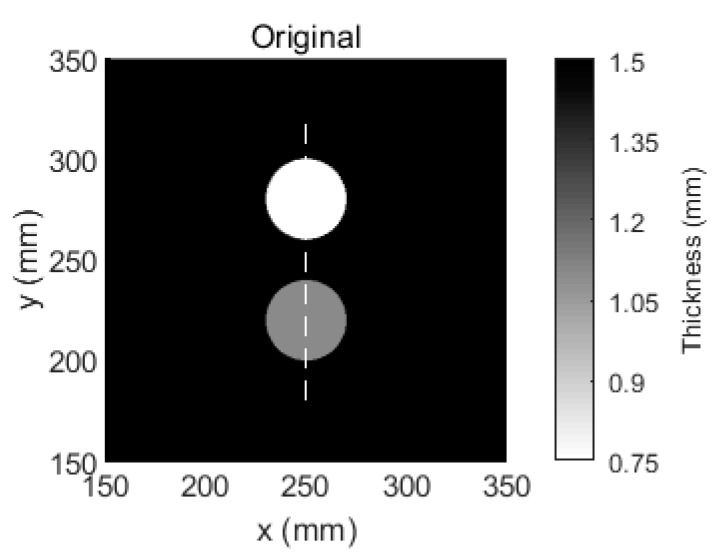
Aluminum plate with a thickness of 1.5 mm and two 40-mm-diameter circular flat-bottomed holes. Their thickness losses are 50% and 30% within the flaws.

**Figure 8:**
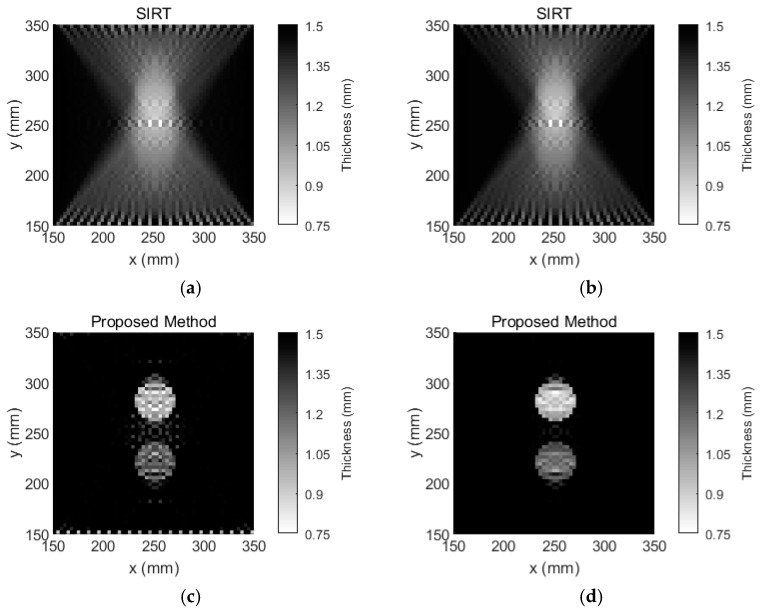
Tomographic reconstruction images for a 1.5-mm-thick aluminum plate with 40-mm-diameter circular flat-bottomed holes. Their thickness losses are 50% and 30% within the flaws. (**a**) The tomographic reconstruction image that is obtained via SIRT without temperature compensation, (**b**) the tomographic reconstruction image that is obtained via SIRT with temperature compensation, (**c**) the tomographic reconstruction image that is obtained via our method without temperature compensation, and (**d**) the tomographic reconstruction image that is obtained via our method with temperature compensation.

**Figure 9:**
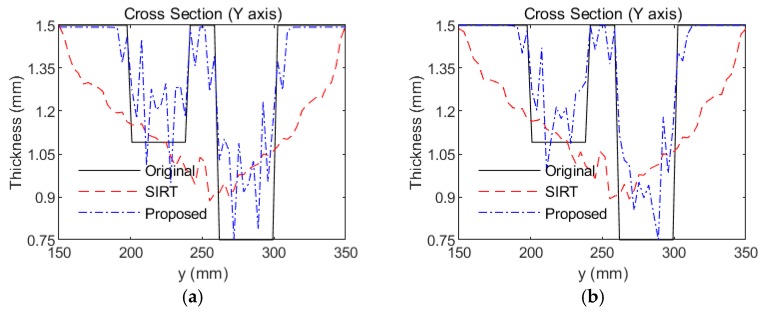
Cross-sections of the reconstructions of two circular flat-bottomed holes along the line that is marked in Figure 7. (**a**) SIRT and our method without temperature compensation and (**b**) SIRT and our method with temperature compensation.

To further verify the performance of the proposed method, an experiment with two regular defects was conducted. Another aluminum plate was used. The baseline temperature in this plate was selected as 25 °C, and the reference temperature was selected as 65 °C. Two 40-mm-diameter circular flat-bottomed holes were made in the same aluminum plate, as shown in Figure 7. The center coordinates of one the holes were x = 250 mm and y = 280 mm, and the thickness loss was 50%. The center coordinates of the remaining hole were x = 250 mm and y = 220 mm, and the thickness loss was 30%. This aluminum plate was placed in the temperature testing box and the temperature was set to 55 °C. That temperature is viewed as the inspection temperature. The tomographic images that were reconstructed using SIRT and our Equation (9) are shown in Figure 8. According to Figure 8a,b the tomographic reconstruction results that were obtained via SIRT confused the two defects with artefacts. In contrast, Figure 8c, which was obtained using the method that is proposed in this paper, clearly shows both thickness losses on the plate, even though temperature compensation was not performed. After temperature compensation, the visible artefacts in the image are no longer observed, as shown in Figure 8d. Moreover, the images of damage that were obtained via our method are more accurate and clearer than those via the SIRT method. The cross-sections of Figure 7 with the damage depths are shown in Figure 9. Our method outperforms SIRT in terms of the depth estimation results. After temperature compensation, the depth estimation accuracy was further improved using our method, as shown in Figure 9b.

**Figure 10:**
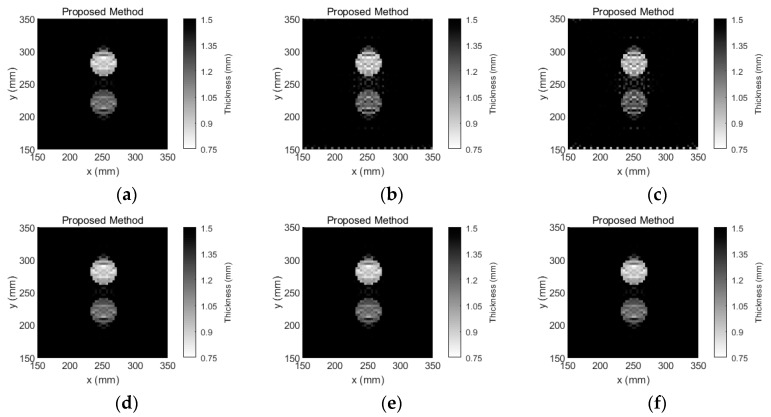
Tomographic reconstruction images for a 1.5-mm-thick aluminum plate with 40-mm-diameter circular flat-bottomed holes. Their thickness losses are 50% and 30% within the flaws. (**a**) The inspection temperature is 25 °C, and temperature compensation is not performed for the tomographic reconstruction image; (**b**) the inspection temperature is 55 °C, and temperature compensation is not performed for the tomographic reconstruction image; (**c**) the inspection temperature is 70 °C, and temperature compensation is not performed for the tomographic reconstruction image; (**d**) the inspection temperature is 25 °C, and temperature compensation is performed for the tomographic reconstruction image; (**e**) the inspection temperature is 55 °C, and temperature compensation is performed for the tomographic reconstruction image; and (**f**) the inspection temperature is 70 °C, and temperature compensation is performed for the tomographic reconstruction image.

The inspection temperatures were set to 25 °C, 55 °C and 70 °C, and the tomographic images that were reconstructed via Equation (9) are shown in Figure 10. According to Figure 10a–c, the number of artefacts significantly increases with the inspection temperature; this occurs because the baseline temperature is 25 °C, and the TOF is changed significantly when the temperature is increased. After temperature compensation, according to Figure 10d–f, the damage image is not influenced by the temperature variation.

## 5. Discussion

The results that are presented in this paper demonstrate that a guided wave that passes through plate-like materials with temperature changes is influenced by the temperature variation. Our approach is not only robust to the temperature variations but also yields more accurate and clearer thickness mappings of plate-like materials compared to SIRT. The visible artefacts in the tomographic reconstruction images are eliminated by our method. The statistical results from Figure 6 and Figure 8 are listed in Table 1. According to Table 1, the number of grids calculated by our method with temperature compensation is almost the same as the area of that number covered by the damage. Meanwhile, since there are many artefacts in the damage image that is reconstructed by the SIRT method, according to Figure 6 and Figure 8, the temperature effect on the SIRT method is not significant. However, from Table 1, the number of artefacts is significantly decreased using the SIRT method after temperature compensation. Moreover, the effectiveness of our temperature compensation method is demonstrated. In addition, the materials that can be inspected by our method are not limited to aluminum plates and the temperature compensation range is not limited to 25 °C to 70 °C. Our method is applicable when the TOF of guided waves that pass through the inspected material is a linear function of the temperature variation. The preliminary experiments are also performed on carbon fiber composites under a temperature variation from 25 °C to 70 °C, and the results demonstrate that the linearity of TOF as a function of temperature with 95% confidence bounds is 0.8563. Hence, the TOF of carbon fiber composites does not have a strong linear relationship with temperature. Therefore, ray tomography for the damage detection of composite materials under temperature variation should be further investigated.

In addition, the computation time of our approach was compared with that of SIRT. The computation times for SIRT and our method in the two experiments are listed in Table 2. The computer that was used in our experiment had an Intel core i7-4770 3.4 GHz quad-core desktop processor with 16 GB of DDR3 memory. According to Table 1, the computational time of our approach is slightly longer than that of SIRT.

The resolution of our method is also limited by the width of the first Fresnel zone, which is expressed as $\sqrt{D\lambda }$, where *λ* is the wavelength of a guided wave and *D* is the distance between the transmitter and receiver transducers. In our experiment, the wavelength of the A0 guided wave mode was approximately 7 mm and the width of the first Fresnel zone was approximately 37 mm. To satisfy ray theory, the sizes of the manufactured defects were much larger than the wavelength, and the distance between the defects exceeded the width of the first Fresnel zone. Guided wave tomography methods have been available for more than 15 years [9]. Compared to a recent paper [18] that was published in Sensors, which also investigates damage monitoring using guided wave tomography, the imaging results of our method have poor resolution. However, our method is robust to temperature variations and has low computational cost. Our method can be used for real-time monitoring with temperature variations. In addition, only isotropic defects of regular shape have been considered in this paper. In the future, we will consider irregular defects and further analyze the accuracy of our method.

## 6. Conclusions

In this paper, we propose a new guided wave ray tomography method that is based on TOF temperature compensation for temperature-robust damage detection. Our approach does not require the estimation of temperature compensation parameters to perform temperature compensation on the guided wave waveform. Our approach only requires the temperature measurement of an inspected material to perform temperature compensation based on the linear relationship between the TOFs of guided waves and the temperature variations. Then, we formulate an optimization problem of the difference between the inspected and baseline TOFs of the guided waves that pass through plate-like materials with temperature variations via an elastic net penalty approach to perform damage mapping of an SHM application via ray tomography. The correctness of our method has been demonstrated experimentally. Additionally, our approach is capable of reconstructing damage mappings of materials without artefacts when the temperature varies from 25 °C to 70 °C. Moreover, the proposed method achieves more accurate and clearer imaging of damage than the SIRT method.

## Figures and Tables

**Table 1:** Number of grids that are covered by the damage area compared to the numbers that are calculated by the SIRT and proposed methods.

Defect	Actual Damage Area	SIRT without T. C. *	SIRT with T. C.	Proposed Method without T. C.	Proposed Method with T. C.
Single defect	177	961	693	236	186
Two defects	224	992	732	332	248

* T. C. represents temperature compensation.

**Table 2:** Computation times of SIRT and our method (UNITS: s).

Defect	SIRT without T. C. *	SIRT with T. C.	Proposed Method without T. C.	Proposed Method with T. C.
Single defect	0.01	0.01	0.89	0.90
Two defects	0.02	0.02	0.97	0.99
